# Long Non-Coding RNAs as Endogenous Target Mimics and Exploration of Their Role in Low Nutrient Stress Tolerance in Plants

**DOI:** 10.3390/genes9090459

**Published:** 2018-09-14

**Authors:** Priyanka Borah, Antara Das, Matthew J. Milner, Arif Ali, Alison R. Bentley, Renu Pandey

**Affiliations:** 1 Mineral Nutrition Laboratory, Division of Plant Physiology, ICAR-Indian Agricultural Research Institute, New Delhi 110 012, India; priyanka.mbbt07@gmail.com; 2 Department of Biosciences, Jamia Millia Islamia, New Delhi 110025, India; aali@jmi.ac.in; 3 ICAR-National Research Centre on Plant Biotechnology, New Delhi 110012, India; antara_10154@iari.res.in; 4 The John Bingham Laboratory, National Institute of Agricultural Botany (NIAB), Huntingdon Road, Cambridge CB30LE, UK; Matthew.Milner@niab.com (M.J.M.), alison.bentley@niab.com (A.R.B.)

**Keywords:** long non-coding RNAs, endogenous target mimicry, microRNA, nitrogen and phosphorus deprivation

## Abstract

Long non-coding RNA (lncRNA) research in plants has recently gained momentum taking cues from studies in animals systems. The availability of next-generation sequencing has enabled genome-wide identification of lncRNA in several plant species. Some lncRNAs are inhibitors of microRNA expression and have a function known as target mimicry with the sequestered transcript known as an endogenous target mimic (eTM). The lncRNAs identified to date show diverse mechanisms of gene regulation, most of which remain poorly understood. In this review, we discuss the role of identified putative lncRNAs that may act as eTMs for nutrient-responsive microRNAs (miRNAs) in plants. If functionally validated, these putative lncRNAs would enhance current understanding of the role of lncRNAs in nutrient homeostasis in plants.

## 1. Introduction

Non-coding RNAs (ncRNAs) are functional RNAs with very low or no potential for encoding protein but are involved in controlling developmental processes or stress responses [1,2]. Non-coding RNAs are a heterogeneous group of RNA molecules that can be classified in different ways according to their location, length and biological functions [3,4]. The first ncRNAs were discovered in the early 1980s when small nuclear RNAs (snRNAs) were established as building blocks of the spliceosome [5].

The application of high throughput RNA-sequencing (hereafter RNAseq) has facilitated the identification of thousands of novel ncRNAs in a diverse range of organisms including human, animals and plants [3,6,7]. In addition to protecting genomes from the introgression of foreign nucleic acids, ncRNAs have been shown to be involved in regulating gene expression at the transcription, RNA processing, and translation levels [8]. The ncRNA-mediated regulation of gene expression at transcriptional, post-transcriptional and epigenetic levels occurs via long non-coding RNAs (lncRNAs), named based on their length relative to microRNAs (miRNAs; around 22 bp in length) [9]. Long non-coding RNAs are large RNAs of length 200 nucleotides or greater and do not code proteins. The diversity of ncRNAs offers an important insight into their dominance in gene regulation. There has been significant work on lncRNAs in the animal kingdom [6,10,11,12,13,14,15,16,17,18,19,20,21,22,23] but studies on plant lncRNA are still in their infancy. There are several intriguing similarities between lncRNAs and messenger RNAs (mRNAs). Similar to mRNAs, most lncRNAs are transcribed by RNA polymerase II, although they can also be transcribed by RNA polymerase III, IV or V [24,25]. A few lncRNAs have features including a 5´ cap and 3´ polyadenylated tail but lack the ability to encode proteins.

In contrast to small RNAs, lncRNA sequences show weak sequence conservation which is proposed as the result of a high rate of primary evolution as the latter may be the frequent target of positive selection [26]. The molecular mechanisms of lncRNAs can be studied by considering lncRNAs in four archetypes: (i) signal (ii) decoys (iii) guides, and (iv) scaffolds [4]. Long non-coding RNAs under the archetype ‘signal’ are transcribed by RNA polymerase II as evidenced by polymerase II occupancy, 5´ caps, histone modification associated with polymerase II transcriptional elongation and polyadenylation [7]. The incorporation of environmental signals such as temperature, light, drought, and salt are some of the well-studied examples of the ‘signal’ type mechanism. Recently reported lncRNA DROUGHT INDUCED lncRNA *(DRIR)* expression under drought and salt stress was observed to regulate the modulation of the activity of genes involved in abiotic stress response in *Arabidopsis thaliana* [27]. DROUGHT INDUCED lncRNA functions upstream of gene transcription whilst its constitutive expression does not affect the expression of other stress responsive genes under normal environmental conditions. Although the molecular mechanism of *DRIR* is still not clear, there is speculation that it might influence the activity or regulation of *FUT4* gene encoding fucosyltransferase, or transcription factor NAC3 (NAM/ATAF/CUC family) or redox status, resulting in enhanced tolerance to drought and salt stress. Another example of an environmental signal mechanism is lncRNA LONG-DAY SPECIFIC MALE FERTILITY ASSOCIATED RNA (*LDMAR*), which causes photoperiod sensitive male sterility in rice, thus regulating fertility by day length [28].

The second ‘decoy’ mechanism demonstrates negative regulation of transcription. Here, the RNA acts as a *molecular sink* and miRNA target mimics are good examples of this archetype. Long non-coding RNAs are thought to act as a decoy and moderate the quantity of protein produced, effectively titrating away proteins and small regulatory RNAs [4]. In silico analysis of maize (*Zea mays*) degradome data showed that there are 86 lncRNAs which act as decoys for 58 miRNA [29]. Their work has confirmed that miRNA decoy sites are conserved across genomes of five monocot species and the putative miRNA decoys can inhibit miRNA function in a spatial or temporal manner [30]. This has been proposed as a contributing factor for maize transcript complexity. A well-studied example of the decoy mechanism is INDUCED BY PHOSPHATE STARVATION 1 (*IPS1*) which acts as a decoy of miRNA399 (further discussed below).

The third ‘guide’ mechanism involves the binding of RNA and positioning of the ribo-nucleo protein complex to a specific target, in either-*cis* or -*trans*, in such a way that cannot be easily predicted based on lncRNA sequences. The Nuclear Speckles RNA-binding protein (NSRs) mediates the alternative splicing of a group of genes involved in lateral root initiation in *A. thaliana*. However, the lncRNA ALTERNATIVE SPLICING COMPETITOR (*ASCO*)-RNA can bind with NSRs and alter alternative splicing resulting in weakening of lateral root initiation [31]. A recent example is ELF18-INDUCED LONG-NONCODING RNA1 (*ELENA1*) lncRNA which is induced by disease caused by *Pseudomonas syringae* and is involved in imparting plant immunity [32]. They provided evidence by using a combination of overexpressed and mutant/knockdown lines of *A. thaliana* showing that *ELENA1* and *MED19a* subunit (a mediator that liaise between transcription factors and RNA Pol II) function interdependently to induce expression of PATHOGENESIS RELATED (*PR1* and *PR2*), β-1,3-glucanases and salicylic acid induced genes. *ELENA1* acts not only in *cis* by affecting nearby locus but also in *trans* by influencing *PR1* and other loci not close to the *ELENA1* locus. 

The final archetype ‘scaffold’ mechanism serves as a central platform upon which relevant molecular components are assembled. A putative example of a scaffold mechanism is COLD ASSISTED INTRONIC NONCODING RNA (*COLDAIR*) involved in vegetative to reproductive transition in *A. thaliana*. *COLDAIR* recruits the polycomb repressive complex 2 (PRC2), a group of proteins that can cause histone modification [33]. During vernalization, increased enrichment of PRC2 causes mitotically stable silencing of the floral repressor gene *FLOWERING LOCUS C* (*FLC*) through chromatin modification. In this process, PRC2 mediates histone H3Lys27 methylation at *FLC* locus causing silencing of *FLC* [34,35]. These examples demonstrate the functional relevance of lncRNAs in plants.

Plants are sessile in nature and must continually evolve their adaptive mechanism to cope with environmental change. Under natural growing conditions, plants are subject to numerous biotic and abiotic stresses including nutrient limitation. Efficient nutrient acquisition is one of the major factors required for sustainable productivity in low input agriculture. Developing nutrient use efficient crops is required in order to minimize nutrient loss and resulting in less environmental pollution, and to reduce the input costs associated with fertilizer application [36,37]. This is particularly pressing for the macronutrient nitrogen (N) and phosphorus (P) which are primary elements required by plants to sustain growth and yield. Nitrogen is the most limiting nutrient, required in large quantities by plants for optimum growth and grain development. Nitrogen and phosphorus are the building blocks of DNA and proteins and about 16% of plant proteins are comprised of N [38]. A number of the lncRNAs discovered show either direct or indirect involvement in nutrient stress adaptation and tolerance in plants [39,40,41]. Further work is required to elucidate the role of lncRNAs as endogenous target mimics of miRNAs and their role in low N and P stress tolerance in plants.

## 2. Role of lncRNAs as Endogenous Target Mimics for MicroRNAs

**Figure 1:**
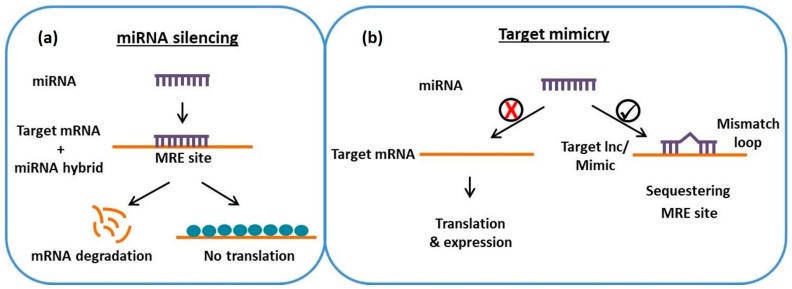
A schematic representation of (**a**) regular miRNA silencing, and (**b**) target mimicry mechanism. miRNA: Micro RNA; mRNA: Messenger RNA; MRE: miRNA recognition elements.

As a negative regulator of mRNA, miRNA has diverse regulatory roles across kingdoms [42]. Among the ncRNAs, miRNA is the most frequently studied ncRNA [43]. It acts in a sequence specific manner to interact with targets via base pairing to complementary sites. This interaction leads to the silencing of specific protein-coding genes at the post-transcriptional level (Figure 1A). This is achieved through the induction of mRNA cleavage accompanied by protein translation. By this mechanism of action, miRNA plays a key regulatory role in diverse developmental processes, stress responses and metabolism [44,45].

In addition to classical protein coding mRNAs, several ncRNAs containing competing miRNA-binding sites are included as targets for miRNAs. It is assumed in existing literature that functional endogenous target mimics (eTMs) are mainly composed of lncRNAs [30,46]. Emergence of lncRNAs as eTMs of miRNAs was first supported by the discovery of *IPS1* in *A. thaliana* [46]. The transcripts of *IPS1* and *PHO2* (*Phosphate 2*) mRNA are key genes involved in inorganic P (Pi) homeostasis, and possess a similar 23 nucleotide (nt) binding site which competes for pairing with miR399. In contrast to the miRNA-binding sites of *PHO2*, the *IPS1* binding site forms a three-nucleotide bulge opposite the miR399 cleavage site on miRNA binding. This bulge prevents the cleavage of *IPS1* and sequesters miR399; thus, the original *PHO2* target is deregulated (Figure 1B). Known as target mimicry, this mechanism of inhibition of miRNA activity results in sequestered transcripts known as competing endogenous RNAs (ceRNAs) [47,48].

In plants, these ceRNAs are known as target mimics (TMs) while in mammals the terms miRNA decoys or microRNA sponges are synonymously used for TMs [49] miRNA targets and TMs have highly similar target sites, known as miRNA recognition elements (MREs). The Franco-Zorrilla method of miRNA/TM interaction is based on MRE conservation and the position of the three-nucleotide bulge [46,47] but later work demonstrated that poor central complementarity is sufficient for the inhibition of miRNA [50,51].

Target mimicry has provided an alternative method for functional studies on miRNAs. As miRNAs have been shown to be regulated up to two-thirds of a eukaryotic transcriptome [52], the relevance of TMs has increased [42,47,53,54]. Target mimicry effects can also be induced by engineered artificial miRNA TMs [30,49]. Further, Short-Tandem Target Mimicry (STTM) technology has been used to engineer artificial target mimics (aTMs) [49]. An example of aTMs designed to establish the functionality of eTMs for miR160c (ath-eTM160-1) and miR166 (ath-eTM166-1) in *A. thaliana* was described by Wu et al. [30] with the resultant transgenic plants overexpressing eTMs displaying distinctly altered phenotypes such as dwarf plant size, serrated leaves and accelerated flowering time as compared to the wild type. These results suggest that eTMs plays a key role in regulating plant developmental processes.

There are various computational methods currently available to help predict TMs in plants. Specifically, TAPIR [55] provides target prediction of plant miRNAs including target mimics for eleven plant genomes. Available databases of plant TMs include the Plant Endogenous Target Mimics database (PeTMbase [56]), miRSponge [57] and the Plant Competing Endogenous RNA database (PceRBase [58]). The main features and links of these databases are listed in Table 1. 

## 3. Long non-coding RNAs Expressed under Nitrogen and Phosphorus Deprivation

Understanding plant response to changes in nutrient status or concentration has advanced significantly [36,37,59,60,61,62,63,64,65,66]. Developed computational approaches can provide integrated views of networks and enhanced understanding of how plants respond to an added nutrient [67]. Additional novel experimental and computational tools in nutrient homeostasis are required to improve nutrient use efficiency in crop plant [68]. To improve N use efficiency (NUE), multiple quantitative trait loci (QTLs) have been identified in the model species *A. thaliana* as well as in crops such as maize, rice, barley and wheat [69,70]. Differential gene expression studies in response to N have also attempted to identify major regulators of expression differences for NUE in wheat [71], sorghum [72], barley [73], maize [40] and rice [74].

By performing ultra-deep sequencing of total RNA, Lvet al. [40] identified intergenic/intronic lncRNAs expressed in maize leaves from plants grown under N deficient and N sufficient conditions. This is the only report in a crop species showing the role of lncRNAs in regulation of the response to N nutrition. Among the 7245 putative lncRNAs identified, approximately 9% (637) were responsive to N, of which 67% (426) were down-regulated under sufficient N, whilst the remaining 211 were upregulated. For functional prediction of differentially expressed lncRNA, co-expression module analysis was performed and functional enrichment suggested their association with processes such as oxidation-reduction, generation of precursor metabolites and energy production [39]. Chen et al. [39] reported an adaptation mechanism to low N stress in the model woody species *Populus tomentosa*. They performed genome-wide identification of functional lncRNAs on *P. tomentosa* plantlets grown with low and sufficient N, and aligned to the genome of *Populus trichocarpa*. They reported 388 unique lncRNA candidates of which seven lncRNAs belonged to seven conserved ncRNA family whilst the majority (381) were novel with no homology in Rfam ncRNA family suggesting that these were specific to *P. tomentosa* species. In response to N deficiency, 126 lncRNAs were differentially expressed, of which 8 were repressed and 118 were induced. Antisense transcripts with interaction with sense genes were found to be involved in plant response to low N stress tolerance. This work is an illustration of the role of lncRNAs and the mechanism of action under N stress tolerance in woody plants. Further identification of antisense lncRNA and their associated regulatory mechanism would increase our understanding of inherent mechanisms to combat low N stress and to determine species specificity. 

In plants, P has essential roles in structural (nucleic acids, phospholipids), metabolic (energy transfer) and regulatory functions (signal transduction) [75]. Phosphorus nutrition influences crop yield by imposing significant effects on above- (leaf area, dry matter accumulation, leaf P content, photosynthesis) and below-ground (root morphological traits, root exudation, symbiosis) processes [37,61,76]. In soils with low P availability, plants adapt by altering their root system architecture [65,77,78,79,80,81], organic acid exudation pattern [82,83,84,85], and can bypass certain metabolic processes [86,87,88,89]. Numerous studies have assessed miRNA directed P_i_ homeostasis in plants identifying miR399, a post-transcriptional regulator, as a major component of P starvation tolerance [59,60,90,91,92]. It serves as a long-distance signal for regulation of plant P_i_ homeostasis [59,60]. Regulation of *PHO2* by miR399 and a ribo-regulator, *At4*, have been shown to be induced by *IPS1* in *A. thaliana* [93]. The members of *IPS1* family have been shown to be ribo-regulators rather than targets of miR399 [59]. The P_i_ starvation responsive miR399 guides the cleavage of *PHO2* RNA, which encodes an E2-ubiquitin conjugase protein that negatively influences shoot P_i_ content and P_i_ remobilization through an unknown mechanism [94]. In *Medicago truncatula* grown under to P stress, three lncRNAs, PHOSPHATE DEFICIENCY INDUCED lncRNA (*PDILs*), were identified and functionally characterized [95]. Of the three, *PDIL1* functions in suppression of degradation of *MtPHO2* while the other two, *PDIL2* and *PDIL3*, are involved at the transcriptional level to regulate P_i_ transport. In addition to *PHO2* mRNA, other ncRNAs containing a region of complementarity with miR399 including *TOMATO PHOSPHATE STARVATION-INDUCED 1* (*TPSI1*) gene were identified in tomato (*Solanum esculentum*) [96], *Mt4* in *M. truncatula* [97,98]. Di et al. [99] reported that many lncRNAs have the potential to regulate mRNA levels in *A. thaliana*. Their extensive study of miR399 targets revealed the mutual interaction of miRNA, lncRNA and mRNAs. For example, under P_i_ and iron homeostasis, At5G01591.1 and *AtFer1* (ferritin) transcripts were induced in response to P_i_ starvation, and their promoter contains Phosphate starvation response 1-Binding Sequence (*P1BS*) motif, indicating that both are also regulated by *PHR1* during P_i_ starvation [100].

Another study in the diatom *Phaeodactylum tricornutum* identified 1510 putative long intergenic ncRNAs (lincRNA) which were responsive to P deficiency. Among these, the same set of 202 lncRNAs was upregulated and downregulated under P stress and P resupply to the growth medium [101]. Two lncRNAs, pti-MIR5472 and pti-MIR5471, were identified as precursors of annotated miRNAs. These lncRNAs were also significantly upregulated in response to P stress in *P. tricornutum* which implies that only these two lncRNAs were common between N and P deficiency stresses. This is also an indication of specificity of lncRNA expression towards a particular stress. 

## 4. Putative Endogenous Target Mimics under Low Nitrogen and Phosphate Stress

We used plant miRBase (http://www.mirbase.org [102]) and retrieved sequences of differentially expressed miRNAs in response to N and P stress [103,104]. These miRNAs were then used to identify their putative target lncRNAs as eTM using PeTMbase (http://www.petmbase.org). PeTMbase is an online resource for endogenous miRNA target mimics for plants which searches the eTMs by corresponding miRNA name or plant species [56]. The identified lncRNAs which may act as eTMs involved in low N and P stress in model (*A. thaliana*) and crop species are summarized in Table 2 along with the corresponding miRNAs. While searching for eTMs, some classes of miRNAs (miR168a, miR171c, miR315b, c, f, miR778, miR2111) had no available data, although the other classes for the same miRNAs possessed corresponding eTMs. In this case, and based on the literature surveyed, it is proposed that these particular miRNAs could have another mechanism of action such as translational inhibition [105]. Moreover, lncRNAs have also been shown to have other mechanisms of epigenetic regulations such as chromatin remodeling [35,106], genomic imprinting [4] other than target mimicry. In Table 2, we observed that for a particular miRNA, there were one to seven eTM IDs because there were different regions of complementarity on an eTM sequence on the basis of corresponding lncRNAs.

**Figure 2:**
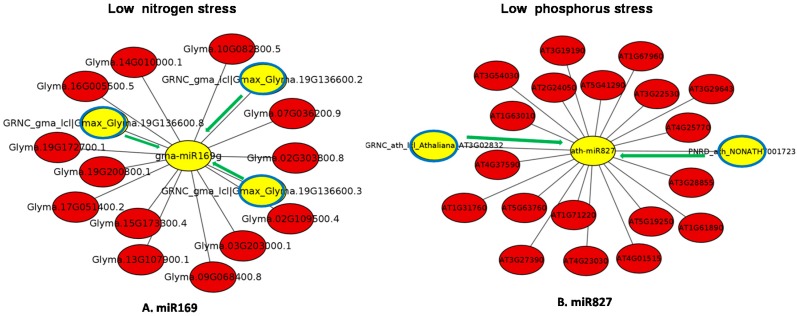
Computationally predicted ‘eTM-miRNA-mRNA’ regulatory network module drawn by selecting a few endogenous target mimics (eTMs) from Table 2 representing low nitrogen (miR169g) and phosphorus (miR827) stress in soybean and Arabidopsis. The central yellow circle is the miRNA while red shows potential targets of the miRNA, and blue circles depict lncRNAs that may act as eTMs. In this gene network, the green arrow shows an interaction between a putative eTM and miRNA while the grey line shows the potential for regulation between miRNAs and their target mRNAs.

To predict the regulatory network module of ‘eTM-miRNA-mRNA’, we selected two miRNAs from Table 2, with one representing stress response to N in soybean and another to P in Arabidopsis (Figure 2). Computational analysis shows that the miR169 (nitrogen regulated) and miR827 (phosphorus regulated) are predicted to be sponged by three and two eTMs, respectively. In soybean, out of 13 potential targets of gma-miR169g (Figure 2A), 11 are involved in encoding different subunits of transcription factor nuclear factor-YA (NF-YA) regulating various developmental processes (GO:0006355) as well as N uptake in plants. The target Glyma.19G172700.1 is involved in regulation of GTPase activity (GO:0043547) while Glyma.02G109500.4 is a hypothetical protein of unknown function. This regulation of miR169 by three eTMs (GRNC_gma_lcl|Gmax_Glyma.19G136600.8, GRNC_gma_lcl|Gmax_Glyma.19G136600.3 and GRNC_gma_lcl|Gmax_Glyma.19G136600.2) in soybean could help the plant indirectly regulate N limiting signalling. Previous work by Zhao et al. [107] has shown that miR169 is down regulated under N starvation and one of its targets, *AtNFYA* is induced both in roots and shoots under limiting N. Overexpression of ath-miR169a led to decreased *AtNFYA* expression leading to lower expression of *AtNRT1.1* and *AtNRT2.1* in both root and shoot and a hypersensitive response to N starvation in the ath miR169aoverexpressing plants compared to wildtype plants [107].

In *Arabidopsis*, 19 potential targets of ath-miR827 (Figure 2B) were identified, the most important being *AT1G63010* involved in maintaining Pi homeostasis (GO:0055062). This gene belongs to the major facilitator superfamily (MFS) and encodes for SPX (SYG1/Pho81/XPR1) domain which is a negative regulator of Pi signalling. SPX proteins repress PHR1/PHR2, thereby regulating Pi starvation induced genes under P stress as reported in rice [108] and *Arabidopsis* [109]. The sequestration of miR827 by eTMs (PNRD_ath_NONATHT001723 and GRNC_ath_lcl|Athaliana_AT3G02832.1) thus could regulate P starvation signaling similar to that seen by ath-miR169 and N. In rice, it has been shown that osa-miR827 is expressed under Pi starvation and targets two genes, SPX-MSF1 and SPX-MFS2, which are under the control of OsPHR1 [110]. While direct evidence of the regulatory mechanism by miR827 SPX and PHR1/PHR2 has not been shown this would suggest that the targets of eTMs are transcription factors which regulate uptake transporters and not the transporters themselves.

Other interesting predicted targets of miR827 were AT4G23030 and AT1G61890 which belong to the multidrug and toxic compound extrusion (MATE) transporter family (GO:0005215). Reports have provided evidence that MATE located at the plasma membrane is involved in organic acid transport efflux under P starvation [89,111]. However, this example predicts a direct regulatory network of eTM-miR827-MATE without involvement of any transcription factor. Other targets of atm-miR827 identified were AT4G37590 which has a role in flower development (GO:0009908) whileAT5G63760 and AT2G24050 are present in the cytoplasm (GO:0005737). AT1G31760 is involved in mitochondrial DNA repair (GO:0043504) while AT4G25770 is involved in chloroplast development (GO:0009507). Target AT3G54030 encodes a protein kinase and functions in ATP binding (GO:0005524). Thus, the miRNAs differentially expressed under low N and P stress have several target genes, but may be sequestered by the lncRNAs (predicted to be eTMs) and regulate metabolic processes and developmental processes. However, functional validation of these lncRNAs for acting as potential eTMs needs to be functionally validated further in order to add to our current understanding of the role of lncRNAs in nutrient homeostasis in plants.

## 5. Conclusions and Future Prospects

Understanding the role of lncRNA in plants is still in its infancy in comparison to progress made in the animal kingdom. Future research is required to systematically identify additional lncRNAs and their role in communicating abiotic and biotic signals in plants. Due to the low expression level of lncRNAs and dependency on specific signals, future work should also focus on the identification of cell type specific lncRNAs [3]. This may be achieved by in-depth transcriptome analysis of single cell or tissue type for lncRNA identification [120]. There is also an opportunity to create a database devoted to stress-responsive lncRNAs. Understanding of the interaction of lncRNAs with other molecular elements in the cell is also an interesting area which needs to be further developed. Although in silico studies have been conducted in plant lncRNA research, functional characterization is still a challenging task, but developments such as advanced imaging for large-scale screening of mutant libraries (e.g., as developed in rice [121]) should accelerate future progress in functional studies of lncRNAs in plants. 

## Figures and Tables

**Table 1:** Summary of databases/webservers used in plant target mimicry research till date.

Name	Features	Links	Reference
TAPIR	Only tool for the TM prediction in plants; applies Franco-Zorrilla rule for target mimicry; contains data for 10 plant species; RNA hybrid and miRBase are the data sources	http://bioinformatics.psb.ugent.be/webtools/tapir/	[55]
miRSponge	1.6% data is from plant and others are of non-plant; experimentally validated; literature mining is the data source	http://www.bio-bigdata.net/miRSponge/	[57]
PeTMbase	Contains 2728 TMs for 11 species; uses Wu et al. (2013) target mimicry rule; GreeNC, PNRD, miRBase, NCBI SRA are the data sources	http://petmbase.org	[56]
PceRBase	First database for plant TMs; 167608 TMs from 26 plant species; Phytozomev10, TAIR10, MSU RGGP & miRBase and literature are the data sources	http://bis.zju.edu.cn/pcernadb/	[58]

TAPIR: Target prediction of plant miRNAs; miRSponge: microRNA sponge; PeTMbase: Plant endogenous target mimics database; PceRBase: Plant competing endogenous RNA database.

**Table 2:** Long non-coding RNAs identified as putative eTM involved in nitrogen and phosphorus deprivation in various plant species [55]. The differentially expressed miRNA sequences specific to nitrogen and phosphorus were retrieved from ‘miRBase’ and employed to find the putative eTMs using ‘PeTMbase’. Arrows ↑— up regulated, ↓— down regulated.

miRNA ID	eTM ID	lncRNA ID	Low N	Low P	References
ath-miR156a-5p	ath_eTM_miR156a-5p-2	GRNC_ath_lcl|Athaliana_AT1G52347.1 gene = AT1G52347		*A. thaliana* (P↑)	[112]
	ath_eTM_miR156a-5p-1	PNRD_ath_NONATHT000580			
ath-miR156b-5p	ath_eTM_miR156b-5p-2	GRNC_ath_lcl|Athaliana_AT1G52347.1 gene = AT1G52347	*A. thaliana* (N↑)		[113]
	ath_eTM_miR156b-5p-1	PNRD_ath_NONATHT000580			
ath-miR156c-5p	ath_eTM_miR156c-5p-2	GRNC_ath_lcl|Athaliana_AT1G52347.1 gene = AT1G52347	*A. thaliana* (N↑)		[113]
	ath_eTM_miR156c-5p-1	PNRD_ath_NONATHT000580			
ath-miR156d-5p	ath_eTM_miR156d-5p-2	GRNC_ath_lcl|Athaliana_AT1G52347.1 gene = AT1G52347	*A. thaliana* (N↑)		[113]
	ath_eTM_miR156d-5p-1	PNRD_ath_NONATHT000580			
ath-miR156e	ath_eTM_miR156e-2	GRNC_ath_lcl|Athaliana_AT1G52347.1 gene = AT1G52347	*A. thaliana* (N↑)		[113]
	ath_eTM_miR156e-1	PNRD_ath_NONATHT000580			
gma-miR156e	gma_eTM_miR156e-3	GRNC_gma_lcl|Gmax_Glyma.18G293400.2		Soybean (P↑)	[114]
	gma_eTM_miR156e-2	GRNC_gma_lcl|Gmax_Glyma.18G293400.1			
	gma_eTM_miR156e-1	GRNC_gma_lcl|Gmax_Glyma.05G242200.1			
ath-miR156f-5p	ath_eTM_miR156f-5p-2	GRNC_ath_lcl|Athaliana_AT1G52347.1 gene = AT1G52347	*A. thaliana* (N↑)		[113]
	ath_eTM_miR156f-5p-1	PNRD_ath_NONATHT000580			
ath-miR156g	ath_eTM_miR156g-2	GRNC_ath_lcl|Athaliana_AT1G52347.1 gene = AT1G52347	*A. thaliana* (N↑)		[113]
	ath_eTM_miR156g-1	PNRD_ath_NONATHT000580			
ath-miR156h	ath_eTM_miR156h-3	GRNC_ath_lcl|Athaliana_AT3G18217.1 gene = AT3G18217	*A. thaliana* (N↑)		[113]
	ath_eTM_miR156h-2	GRNC_ath_lcl|Athaliana_AT1G52347.1 gene = AT1G52347			
	ath_eTM_miR156h-1	PNRD_ath_NONATHT000580			
gma-miR159a-3p	gma_eTM_miR159a-3p-1	gma_TCONS_00088249		Soybean (P↑)	[114]
tae-miR159b	tae_eTM_miR159b-2	GRNC_tae_lcl|Taestivum_Traes_2DS_9A9CAF0B0.1		Wheat(P↑)	[115]
	tae_eTM_miR159b-1	GRNC_tae_lcl|Taestivum_Traes_1AL_8C8E43898.1			
zma-miR160a-3p	zma_eTM_miR160a-3p-1	GRNC_zma_lcl|Zmays_AC211588.3_FGT002	Maize (N↑)		[116]
zma-miR160a-5p	zma_eTM_miR160a-5p-5	GRNC_zma_lcl|Zmays_GRMZM5G849473_T01	Maize (N↑)		[116]
	zma_eTM_miR160a-5p-4	GRNC_zma_lcl|Zmays_GRMZM2G531719_T01			
	zma_eTM_miR160a-5p-3	GRNC_zma_lcl|Zmays_GRMZM2G149698_T05			
	zma_eTM_miR160a-5p-2	GRNC_zma_lcl|Zmays_GRMZM2G011007_T01			
	zma_eTM_miR160a-5p-1	PNRD_zma_GRMZM5G849473_T01			
zma-miR160b-5p	zma_eTM_miR160b-5p-5	GRNC_zma_lcl|Zmays_GRMZM5G849473_T01	Maize (N↑)		[116]
	zma_eTM_miR160b-5p-4	GRNC_zma_lcl|Zmays_GRMZM2G531719_T01			
	zma_eTM_miR160b-5p-3	GRNC_zma_lcl|Zmays_GRMZM2G149698_T05			
	zma_eTM_miR160b-5p-2	GRNC_zma_lcl|Zmays_GRMZM2G011007_T01			
	zma_eTM_miR160b-5p-1	PNRD_zma_GRMZM5G849473_T01			
zma-miR160c-5p	zma_eTM_miR160c-5p-5	GRNC_zma_lcl|Zmays_GRMZM5G849473_T01	Maize (N↑)		[116]
	zma_eTM_miR160c-5p-4	GRNC_zma_lcl|Zmays_GRMZM2G531719_T01			
	zma_eTM_miR160c-5p-3	GRNC_zma_lcl|Zmays_GRMZM2G149698_T05			
	zma_eTM_miR160c-5p-2	GRNC_zma_lcl|Zmays_GRMZM2G011007_T01			
	zma_eTM_miR160c-5p-1	PNRD_zma_GRMZM5G849473_T01			
zma-miR160d-3p	zma_eTM_miR160d-3p-3	GRNC_zma_lcl|Zmays_GRMZM2G064666_T02	Maize (N↑)		[113]
	zma_eTM_miR160d-3p-2	GRNC_zma_lcl|Zmays_GRMZM2G054392_T01			
	zma_eTM_miR160d-3p-1	GRNC_zma_lcl|Zmays_GRMZM2G052412_T01			
zma-miR160d-5p	zma_eTM_miR160d-5p-5	GRNC_zma_lcl|Zmays_GRMZM5G849473_T01	Maize (N↑)		[116]
	zma_eTM_miR160d-5p-4	GRNC_zma_lcl|Zmays_GRMZM2G531719_T01			
	zma_eTM_miR160d-5p-3	GRNC_zma_lcl|Zmays_GRMZM2G149698_T05			
	zma_eTM_miR160d-5p-2	GRNC_zma_lcl|Zmays_GRMZM2G011007_T01			
	zma_eTM_miR160d-5p-1	PNRD_zma_GRMZM5G849473_T01			
zma-miR160e	zma_eTM_miR160e-5	GRNC_zma_lcl|Zmays_GRMZM5G849473_T01	Maize (N↑)		[116]
	zma_eTM_miR160e-4	GRNC_zma_lcl|Zmays_GRMZM2G531719_T01			
	zma_eTM_miR160e-3	GRNC_zma_lcl|Zmays_GRMZM2G149698_T05			
	zma_eTM_miR160e-2	GRNC_zma_lcl|Zmays_GRMZM2G011007_T01			
	zma_eTM_miR160e-1	PNRD_zma_GRMZM5G849473_T01			
zma-miR160g-5p	zma_eTM_miR160g-5p-5	GRNC_zma_lcl|Zmays_GRMZM5G849473_T01	Maize (N↑)		[116]
	zma_eTM_miR160g-5p-4	GRNC_zma_lcl|Zmays_GRMZM2G531719_T01			
	zma_eTM_miR160g-5p-3	GRNC_zma_lcl|Zmays_GRMZM2G149698_T05			
	zma_eTM_miR160g-5p-2	GRNC_zma_lcl|Zmays_GRMZM2G011007_T01			
	zma_eTM_miR160g-5p-1	PNRD_zma_GRMZM5G849473_T01			
zma-miR164f-5p	zma_eTM_miR164f-5p-1	GRNC_zma_lcl|Zmays_GRMZM2G008252_T01	Maize (N↑)		[116]
zma-miR164f-3p	zma_eTM_miR164f-3p-1	GRNC_zma_lcl|Zmays_GRMZM5G837428_T01	Maize (N↑)		[113]
zma-miR166j-3p	zma_eTM_miR166j-3p-4	GRNC_zma_lcl|Zmays_GRMZM2G134604_T01	Maize (N↓)		[117]
	zma_eTM_miR166j-3p-3	zma_eTM_miR166j-3p-3			
	zma_eTM_miR166j-3p-2	zma_eTM_miR166j-3p-2			
	zma_eTM_miR166j-3p-1	zma_TCONS_00089106			
zma-miR166k-3p	zma_eTM_miR166k-3p-4	GRNC_zma_lcl|Zmays_GRMZM2G134604_T01	Maize (N↓)		[117]
	zma_eTM_miR166k-3p-3	GRNC_zma_lcl|Zmays_GRMZM2G110279_T02			
	zma_eTM_miR166k-3p-2	GRNC_zma_lcl|Zmays_GRMZM2G110279_T01			
	zma_eTM_miR166k-3p-1	zma_TCONS_00089106			
zma-miR166n-3p	zma_eTM_miR166n-3p-4	GRNC_zma_lcl|Zmays_GRMZM2G134604_T01	Maize (N↓)		[117]
	zma_eTM_miR166n-3p-3	GRNC_zma_lcl|Zmays_GRMZM2G110279_T02			
	zma_eTM_miR166n-3p-2	GRNC_zma_lcl|Zmays_GRMZM2G110279_T01			
	zma_eTM_miR166n-3p-1	zma_TCONS_00089106			
zma-miR167g-3p	zma_eTM_miR167g-3p-1	zma_TCONS_00081049	Maize (N↓)		[116]
zma-miR167g-5p	zma_eTM_miR167g-5p-2	GRNC_zma_lcl|Zmays_GRMZM2G174168_T02	Maize (N↓)		[116]
	zma_eTM_miR167g-5p-1	GRNC_zma_lcl|Zmays_GRMZM2G174168_T01			
zma-miR167h-3p	zma_eTM_miR167h-3p-7	GRNC_zma_lcl|Zmays_GRMZM2G326635_T01	Maize (N↓)		[116]
	zma_eTM_miR167h-3p-6	GRNC_zma_lcl|Zmays_GRMZM2G175272_T03			
	zma_eTM_miR167h-3p-5	GRNC_zma_lcl|Zmays_GRMZM2G159741_T04			
	zma_eTM_miR167h-3p-4	GRNC_zma_lcl|Zmays_GRMZM2G158766_T04			
	zma_eTM_miR167h-3p-3	GRNC_zma_lcl|Zmays_GRMZM2G125239_T04			
	zma_eTM_miR167h-3p-2	PNRD_zma_TCONS_00034773			
	zma_eTM_miR167h-3p-1	zma_TCONS_00012947			
zma-miR167h-5p	zma_eTM_miR167h-5p-2	GRNC_zma_lcl|Zmays_GRMZM2G174168_T02	Maize (N↓)		[116]
	zma_eTM_miR167h-5p-1	GRNC_zma_lcl|Zmays_GRMZM2G174168_T01			
gma-miR169f	gma_eTM_miR169f-3	GRNC_gma_lcl|Gmax_Glyma.19G136600.8	Soybean (N↑)		[118]
	gma_eTM_miR169f-2	GRNC_gma_lcl|Gmax_Glyma.19G136600.3			
	gma_eTM_miR169f-1	GRNC_gma_lcl|Gmax_Glyma.19G136600.2			
gma-miR169g	gma_eTM_miR169g-3	GRNC_gma_lcl|Gmax_Glyma.19G136600.8	Soybean (N↑)		[118]
	gma_eTM_miR169g-2	GRNC_gma_lcl|Gmax_Glyma.19G136600.3			
	gma_eTM_miR169g-1	GRNC_gma_lcl|Gmax_Glyma.19G136600.2			
ath-miR169a-3p	ath_eTM_miR169a-3p-2	GRNC_ath_lcl|Athaliana_AT1G44940.2		*A. thaliana*(P↓)	[113]
	ath_eTM_miR169a-3p-1	GRNC_ath_lcl|Athaliana_AT1G44940.1			
zma-miR319a-5p	zma_eTM_miR319a-5p-4	GRNC_zma_lcl|Zmays_GRMZM2G438722_T03	Maize (N↑)		[116]
	zma_eTM_miR319a-5p-3	zma_TCONS_00089764			
	zma_eTM_miR319a-5p-2	zma_TCONS_00089763			
	zma_eTM_miR319a-5p-1	zma_TCONS_00024738			
zma-miR395d-5p	zma_eTM_miR395d-5p-1	zma_TCONS_00091080	Maize (N↓)		[116]
zma-miR395g-5p	zma_eTM_miR395g-5p-1	zma_TCONS_00091080			
gma-miR398b	gma_eTM_miR398b-1	GRNC_gma_lcl|Gmax_Glyma.12G204100.1		Soybean (P↓)	[114]
ath-miR399f	ath_eTM_miR399f-5	GRNC_ath_lcl|Athaliana_AT5G03545.1	*A. thaliana* (N↑↓)	*A. thaliana* (P↑)	[113]
	ath_eTM_miR399f-4	GRNC_ath_lcl|Athaliana_AT3G09922.1			
	ath_eTM_miR399f-3	PNRD_ath_At4-2			
ath-miR399e	ath_eTM_miR399f-2	PNRD_ath_At4			
	ath_eTM_miR399f-1	PNRD_ath_AtIPS1			
	ath_eTM_miR399e-1	GRNC_ath_lcl|Athaliana_AT1G53708.1	*A. thaliana* (N↑↓)	*A. thaliana* (P↑)	[113]
ath-miR399d	ath_eTM_miR399d-5	GRNC_ath_lcl|Athaliana_AT5G03545.1	*A. thaliana* (N↑↓)	*A. thaliana* (P↑)	[113]
	ath_eTM_miR399d-4	GRNC_ath_lcl|Athaliana_AT3G09922.1			
	ath_eTM_miR399d-3	PNRD_ath_At4-2			
ath-miR399b	ath_eTM_miR399d-2	PNRD_ath_At4			
	ath_eTM_miR399d-1	PNRD_ath_AtIPS1			
	ath_eTM_miR399b-6	GRNC_ath_lcl|Athaliana_AT5G03545.1	*A. thaliana* (N↑↓)	*A. thaliana* (P↑)	[113]
	ath_eTM_miR399b-5	GRNC_ath_lcl|Athaliana_AT3G09922.1			
	ath_eTM_miR399b-4	GRNC_ath_lcl|Athaliana_AT1G53708.1			
ath-miR399a	ath_eTM_miR399b-3	PNRD_ath_At4-2			
	ath_eTM_miR399b-2	PNRD_ath_At4			
	ath_eTM_miR399b-1	PNRD_ath_AtIPS1			
	ath_eTM_miR399a-6	GRNC_ath_lcl|Athaliana_AT5G03545.1	*A. thaliana* (N↑↓)	*A. thaliana* (P↑)	[113]
	ath_eTM_miR399a-5	GRNC_ath_lcl|Athaliana_AT3G09922.1			
	ath_eTM_miR399a-4	GRNC_ath_lcl|Athaliana_AT1G53708.1			
	ath_eTM_miR399a-3	PNRD_ath_At4-2			
	ath_eTM_miR399a-2	PNRD_ath_At4			
	ath_eTM_miR399a-1	PNRD_ath_AtIPS1			
tae-miR408	tae_eTM_miR408-2	GRNC_tae_lcl|Taestivum_Traes_5BL_EED36D3B9.35		Wheat (P↓)	[115]
	tae_eTM_miR408-1	tae_TCONS_00103503			
osa-miR444a-3p.2	osa_eTM_miR444a-3p.2-1	GRNC_osa_lcl|Osativa_LOC_Os12g19080.1	Rice((N↑)		[119]
ath-miR827	ath_eTM_miR827-2	GRNC_ath_lcl|Athaliana_AT3G02832.1 gene = AT3G02832		*A. thaliana* (P↑)	[112]
	ath_eTM_miR827-1	PNRD_ath_NONATHT001723			
ath-miR828	ath_eTM_miR828-1	PNRD_ath_NONATHT000094		*A. thaliana* (P↑)	[112]

## References

[1] Erdmann V.A., Barciszewska M.Z., Szymanski M., Hochberg A., de Groot N., Barciszewski J. (2001). The non-coding RNAs as riboregulators. Nucleic Acids Res..

[2] Wang J., Meng X., Dobrovolskaya O.B., Orlov Y.L., Chen M. (2017). Non-coding RNAs and their roles in stress response in plants. Genom. Proteom. Bioinform..

[3] Liu J., Jung C., Xu J., Wang H., Deng S., Bernad L., Arenas-Huertero C., Chua N.H. (2012). Genome-wide analysis uncovers regulation of long intergenic noncoding RNAs in Arabidopsis. Plant Cell.

[4] Wang K.C., Chang H.Y. (2011). Molecular mechanisms of long noncoding RNAs. Mol. Cell.

[5] Liu X., Hao L., Li D., Zhu L., Hu S. (2015). Long non-coding RNAs and their biological roles in plants. Genom. Proteom. Bioinform..

[6] Ravasi T., Suzuki H., Pang K.C., Katayama S., Furuno M., Okunishi R., Fukuda S., Ru K., Frith M.C., Gongora M.M. (2006). Experimental validation of the regulated expression of large numbers of non-coding RNAs from the mouse genome. Genome Res..

[7] Guttman M., Amit I., Garber M., French C., Lin M.F., Feldser D., Huarte M., Zuk O., Carey B.W., Cassady J.P. (2009). Chromatin signature reveals over a thousand highly conserved large non-coding RNAs in mammals. Nature.

[8] Kung J.T.Y., Colognori D., Lee J.T. (2013). Long noncoding RNAs: Past, present, and future. Genetics.

[9] Wapinski O., Chang H.Y. (2011). Long noncoding RNAs and human disease. Trends Cell Biol..

[10] Brannan C.I., Dees E.C., Ingram R.S., Tilghman S.M. (1990). The product of the H19 gene may function as an RNA. Mol. Cell Biol..

[11] Brown C.J., Lafreniere R.G., Powers V.E., Sebastio G., Ballabio A., Pettigrew A.L., Ledbetter D.H., Levy E., Craig I.W., Willard H.F. (1991). Localization of the X inactivation centre on the human X chromosome in Xq13. Nature.

[12] Bernstein E., Allis C.D. (2005). RNA meets chromatin. Gene. Dev..

[13] Chaumeil J., Le Baccon P., Wutz A., Heard E. (2006). A novel role for *Xist* RNA in the formation of a repressive nuclear compartment into which genes are recruited when silenced. Genes Dev..

[14] Duret L., Chureau C., Samain S., Weissenbach J., Avner P. (2006). The *Xist* RNA gene evolved in eutherians by pseudogenization of a protein-coding gene. Science.

[15] Kapranov P., Cheng J., Dike S., Nix D.A., Duttagupta R., Willingham A.T., Stadler P.F., Hertel J., Hackermuller J., Hofacker I.L. (2007). RNA maps reveal new RNA classes and a possible function for pervasive transcription. Science.

[16] Rinn J.L., Kertesz M., Wang J.K., Squazzo S.L., Xu X., Brugmann S.A., Goodnough L.H., Helms J.A., Farnham P.J., Segal E. (2007). Functional demarcation of active and silent chromatin domains in human HOX loci by noncoding RNAs. Cell.

[17] Mercer T.R., Dinger M.E., Sunkin S.M., Mehler M.F., Mattick J.S. (2008). Specific expression of long noncoding RNAs in the mouse brain. Proc. Natl. Acad. Sci. USA.

[18] Zhao J., Sun B.K., Erwin J.A., Song J.J., Lee J.T. (2008). Polycomb proteins targeted by a short repeat RNA to the mouse X chromosome. Science.

[19] Nagano T., Mitchell J.A., Sanz L.A., Pauler F.M., Ferguson-Smith A.C., Feil R., Fraser P. (2008). The Air noncoding RNA epigenetically silences transcription by targeting G9a to chromatin. Science.

[20] Tsai M.C., Manor O., Wan Y., Mosammaparast N., Wang J.K., Lan F., Shi Y., Segal E., Chang H.Y. (2010). Long noncoding RNA as modular scaffold of histone modification complexes. Science.

[21] Kaneko S., Li G., Son J., Xu C.F., Margueron R., Neubert T.A., Reinberg D. (2010). Phosphorylation of the PRC2 component Ezh2 is cell cycle-regulated and up-regulates its binding to ncRNA. Genes Dev..

[22] Dinger M.E., Pang K.C., Mercer T.R., Crowe M.L., Grimmond S.M., Mattick J.S. (2009). NRED: A database of long noncoding RNA expression. Nucleic Acids Res..

[23] Zhuang L.K., Yang Y.T., Ma X., Han B., Wang Z.S., Zhao Q.Y., Wu L.Q., Qu Z.Q. (2016). MicroRNA-92b promotes hepatocellular carcinoma progression by targeting Smad7 and is mediated by long non-coding RNA XIST. Cell Death Disease.

[24] Haag J.R., Pikaard C.S. (2011). Multisubunit RNA polymerases IV and V: Purveyors of non-coding RNA for plant gene silencing. Nat. Rev. Mol. Cell Biol..

[25] Wu J., Okada T., Fukushima T., Tsudzuki T., Sugiura M., Yukawa Y. (2012). A novel hypoxic stress-responsive long non-coding RNA transcribed by RNA polymerase III in Arabidopsis. RNA Biol..

[26] Pang K.C., Frithand M.C., Mattick J.S. (2006). Rapid evolution of noncoding RNAs: lack of conservation does not mean lack of function. Trends Genet..

[27] Qin T., Zhao H., Cui P., Albesher N., Xiong L. (2017). A nucleus-localized long non-coding RNA enhances drought and salt stress tolerance. Plant Physiol..

[28] Ding J., Lu Q., Ouyang Y., Mao H., Zhang P., Yao J., Xu C., Li X., Xiao J., Zhang Q. (2012). A long noncoding RNA regulates photoperiod-sensitive male sterility, an essential component of hybrid rice. Proc. Natl. Acad. Sci. USA.

[29] Fan C., Hao Z., Yan J., Li G. (2015). Genome-wide identification and functional analysis of lincRNAs acting as miRNA targets or decoys in maize. BMC Genomics.

[30] Wu H.-J., Wang Z.-M., Wang M., Wang X.-J. (2013). Widespread long noncoding RNAs as endogenous target mimics for microRNAs in plants. Plant Physiol..

[31] Bardou F., Ariel F., Simpson C.G., Romero-Barrios N., Laporte P., Balzergue S., Brown J.W.S., Crespi M. (2014). Long noncoding RNA modulates alternative splicing regulators in Arabidopsis. Dev. Cell.

[32] Seo J.S., Sun H.-X., Park B.S., Huang C.-H., Yeh S.-D., Jung C., Chua N.-H. (2017). ELF18-INDUCED LONG-NONCODING RNA associates with mediator to enhance expression of innate immune response genes in Arabidopsis. Plant Cell.

[33] Heo J.B., Sung S. (2011). Vernalization-mediated epigenetic silencing by a long intronic noncoding RNA. Science.

[34] Gendall A.R., Levy Y.Y., Wilson A., Dean C. (2001). The VERNALIZATION 2 gene mediates the epigenetic regulation of vernalization in Arabidopsis. Cell.

[35] Wood C.C., Robertson M., Tanner G., Peacock W.J., Dennis E.S., Helliwell C.A. (2006). The *Arabidopsis thaliana* vernalization response requires a polycomb-like protein complex that also includes VERNALIZATION INSENSITIVE3. Proc. Natl. Acad. Sci. USA.

[36] Nischal L., Mohsin M., Khan I., Kardam H., Wadhwa A., Abrol Y.P., Iqbal M., Ahmad A. (2012). Identification and comparative analysis of microRNAs associated with low-N tolerance in rice genotypes. PLoS ONE.

[37] Elanchezhian R., Krishnapriya V., Pandey R., Rao A.S., Abrol Y.P. (2015). Physiological and molecular approaches for improving phosphorus uptake efficiency of crops. Curr. Sci. India.

[38] Frink C.R., Waggoner P.E., Ausubel J.H. (1999). Nitrogen fertilizer: retrospect and prospect. Proc. Natl. Acad. Sci. USA.

[39] Chen M., Wang C., Bao H., Chen H., Wang Y. (2016). Genome-wide identification and characterization of novel lncRNAs in Populus under nitrogen deficiency. Mol. Genet. Genomics.

[40] Lv Y., Liang Z., Ge M., Qi W., Zhang T., Lin F., Peng Z., Zhao H. (2016). Genome-wide identification and functional prediction of nitrogen-responsive intergenic and intronic long non-coding RNAs in maize (*Zea mays* L.). BMC Genomics.

[41] Amaral N.S.D., Melo N.C., Maia B.d.M., Rocha R.M. (2017). Noncoding RNA profiles in tobacco- and alcohol-associated diseases. Genes.

[42] Steinkraus B.R., Toegel M., Fulga T.A. (2016). Tiny giants of gene regulation: Experimental strategies for microRNA functional studies. Wiley Interdiscip. Rev. Dev. Biol..

[43] Dogini D.B., Pascoal V.D.B., Avansini S.H., Vieira A.S., Pereira T.C., Lopes-Cendes I. (2014). The new world of RNAs. Genet. Mol. Biol..

[44] Lozada-Chávez I., Stadler P.F., Prohaska S.J. (2011). Hypothesis for the modern RNA world: A *pervasive* non-coding RNA-based genetic regulation is a prerequisite for the emergence of multicellular complexity. Orig. Life Evol. Biosph..

[45] Ameres S.L., Zamore P.D. (2013). Diversifying microRNA sequence and function. Nat. Rev. Mol. Cell Biol..

[46] Franco-Zorrilla J.M., Valli A., Todesco M., Mateos I., Puga M.I., Rubio-Somoza I., Leyva A., Weigel D., García J.A., Paz-Ares J. (2007). Target mimicry provides a new mechanism for regulation of microRNA activity. Nat. Genet..

[47] Salmena L., Poliseno L., Tay Y., Kats L., Pandolfi P.P. (2011). A ceRNA hypothesis: The Rosetta stone of a hidden RNA language?. Cell.

[48] Kartha R.V., Subramanian S. (2014). Competing endogenous RNAs (ceRNAs): New entrants to the intricacies of gene regulation. Front. Genet..

[49] Todesco M., Rubio-Somoza I., Paz-Ares J., Weigel D. (2010). A collection of target mimics for comprehensive analysis of MicroRNA function in *Arabidopsis thaliana*. PLoS Genet..

[50] Reichel M., Li Y., Li J., Millar A.A. (2015). Inhibiting plant microRNA activity: Molecular SPONGEs, target MIMICs and STTMs all display variable efficacies against target microRNAs. Plant Biotechnol. J..

[51] Liu Q., Wang F., Axtell M.J. (2014). Analysis of complementarity requirements for plant microRNA targeting using a *Nicotiana benthamiana* quantitative transient assay. Plant Cell.

[52] Strachan T., Read A.P. (2011). Human Molecular Genetics.

[53] Quek X.C., Thomson D.W., Maag J.L.V., Bartonicek N., Signal B., Clark M.B., Gloss B.S., Dinger M.E. (2014). lncRNAdb v2.0: Expanding the reference database for functional long noncoding RNAs. Nucleic Acids Res..

[54] Gupta P.K. (2015). MicroRNAs and target mimics for crop improvement. Curr. Sci. India.

[55] Bonnet E., He Y., Billiau K., Van de Peer Y. (2010). TAPIR, a web server for the prediction of plant microRNA targets, including target mimics. Bioinformatics.

[56] Karakulah G., Yucebilgili-Kurtoglu K., Unver T. (2016). PeTMbase: A Database of Plant Endogenous Target Mimics (eTMs). PLoS ONE.

[57] Wang P., Zhi H., Zhang Y., Liu Y., Zhang J., Gao Y., Guo M., Ning S., Li X. (2015). miRSponge: A manually curated database for experimentally supported miRNA sponges and ceRNAs. Database (Oxford).

[58] Yuan C., Meng X., Li X., Illing N., Ingle R.A., Wang J., Chen M. (2017). PceRBase: A database of plant competing endogenous RNA. Nucleic Acids Res..

[59] Bari R., Pant B.D., Stitt M., Scheible W.R. (2006). PHO2, MicroRNA399, and PHR1 define a phosphate-signaling pathway in plants. Plant Physiol..

[60] Pant B.D., Buhtz A., Kehr J., Scheible W.R. (2008). MicroRNA399 is a long-distance signal for the regulation of plant phosphate homeostasis. Plant J..

[61] López-Arredondo D.L., Leyva-Gonzalez M.A., Gonzalez-Morales S.I., Lopez-Bucio J., Herrera-Estrella L. (2014). Phosphatenutrition: Improvinglow-phosphatetolerance in crops. Annu. Rev. Plant Biol..

[62] Vidal E.A., Moyano T.C., Canales J., Gutiérrez R.A. (2014). Nitrogen control of developmental phase transitions in *Arabidopsis thaliana*. J. Exp. Bot..

[63] Alvarez J.M., Riveras E., Vidal E.A., Gras D.E., Contreras-López O., Tamayo K.P., Aceituno F., Gómez I., Ruffel S., LejayChandra L. (2014). Systems approach identifies TGA1 and TGA4 transcription factors as important regulatory components of the nitrate response of *Arabidopsis thaliana* roots. Plant J..

[64] Ganie A.H., Ahmad A., Pandey R., Aref I.M., Yousuf P.Y., Ahmad S., Iqbal M. (2015). Metabolite profiling of low-P tolerant and low-P sensitive maize genotypes under phosphorus starvation and restoration conditions. PLoS ONE.

[65] Nazir M., Pandey R., Siddiqi T.O., Ibrahim M.M., Qureshi I.M., Vengavasi K., Abraham G., Ahmad A. (2016). Nitrogen-deficiency stress induces protein expression differentially in low-N tolerant and low-N sensitive maize genotypes. Front. Plant Sci..

[66] Giri J., Bhosale R., Huang G., Pandey B.K., Parker H., Zappala S., Yang J., Dievart A., Bureau C., Ljung K. (2018). Rice auxin influx carrier OsAUX1 facilitates root hair elongation in response to low external phosphate. Nat. Commun..

[67] Gutierrez R.A. (2012). Systems biology for enhanced plant nitrogen nutrition. Science.

[68] Ehrhardt D.W., Frommer W.B. (2012). New Technologies for 21st Century Plant Science. Plant Cell.

[69] Agrama H.A.S., Zakaria A.G., Said F.B., Tuinstra M. (1999). Identification of quantitative trait loci for nitrogen use efficiency in maize. Mol. Breeding.

[70] Garnett T., Conn V., Kaiser B.N. (2009). Root based approaches to improving nitrogen use efficiency in plants. Plant Cell Environ..

[71] Curci P.L., AieseCigliano R., Zuluaga D.L., Janni M., Sanseverino W., Sonnante G. (2017). Transcriptomic response of durum wheat to nitrogen starvation. Sci. Rep..

[72] Gelli M., Duo Y., Konda A.R., Zhang C., Holding D., Dweikat I. (2014). Identification of differentially expressed genes between sorghum genotypes with contrasting nitrogen stress tolerance by genome-wide transcriptional profiling. BMC Genomics.

[73] Quan X., Zeng J., Ye L., Chen G., Han Z., Shah J.M., Zhang G. (2016). Transcriptome profiling analysis for two Tibetan wild barley genotypes in responses to low nitrogen. BMC Plant Biol..

[74] Sinha S.K., Amitha Mithra S.V., Chaudhary S., Tyagi P., Venkadesan S., Rani M., Mandal P.K. (2018). Transcriptome analysis of two rice varieties contrasting for nitrogen use efficiency under chronic N starvation reveals differences in chloroplast and starch metabolism-related genes. Genes.

[75] Marschner H., Marschner P. (2012). Mineral Nutrition of Higher Plants.

[76] Pandey R., Zinta G., AbdElgawad H., Ahmad A., Jain V., Janssens I.A. (2015). Physiological and molecular alterations in plants exposed to high [CO_2_] under phosphorus stress. Biotechnol. Adv..

[77] López-Bucio J., Herńandez-Abreu E., Sánchez-Calderón L., Nieto-Jacobo M.F., Simpson J., Herrera-Estrella L. (2002). Phosphate availability alters architecture and causes changes hormone sensitivity in the Arabidopsis root system. Plant Physiol..

[78] Nacry P., Canivenc G., Muller B., Azmi A., Van Onckelen V., Rossignol M., Doumas P. (2005). A role for auxin redistribution in the responses of the root system architecture to phosphate starvation in *Arabidopsis*. Plant Physiol..

[79] Sánchez-Calderón L., López-Bucio J., Chacón-López A., Cruz-Ramírez A., Nieto-Jacobo F., Dubrovsky J.G., Herrera-Estrella J. (2006). Phosphate starvation induces a determinate developmental program in the roots of *Arabidopsis thaliana*. Plant Cell Physiol..

[80] Svistoonoff S., Creff A., Reymond M., Sigoillot-Claude C., Ricaud L., Blanchet A., Nussaume L., Desnos T. (2007). Root tip contact with low-phosphate media reprograms plant root architecture. Nat. Genet..

[81] Wang Y.-L., Almvik M., Clarke N., Eich-Greatorex S., Øgaard A.F., Krogstad T., Lambers H., Clarke J.L. (2015). Contrasting responses of root morphology and root-exuded organic acids to low phosphorus availability in three important food crops with divergent root traits. AoB Plants.

[82] Vengavasi K., Pandey R. (2016). Root exudation index: Screening organic acid exudation and phosphorus acquisition efficiency in soybean genotypes. Crop Pasture Sci..

[83] Vengavasi K., Pandey R., Abraham G., Yadav R.K. (2017). Comparative analysis of soybean root proteome reveals molecular basis of differential carboxylate efflux under low phosphorus stress. Genes.

[84] Wang Y., Krogstad T., Clarke N., Øgaard A.F., Clarke J.L. (2017). Impact of phosphorus on rhizosphere organic anions of wheat at different growth stages under field conditions. AoB Plants.

[85] Vengavasi K., Pandey R. (2018). Root exudation potential in contrasting soybean genotypes in response to low soil phosphorus availability is determined by photo-biochemical processes. Plant Physiol. Biochem..

[86] Palma D.A., Blumwald E., Plaxton W.C. (2000). Upregulation of vascular H^+^-translocating pyrophosphatase by phosphate starvation of *Brassica napus* (rapeseed) suspension cell cultures. FEBS Lett..

[87] Theodorou M.E., Plaxton W.C. (1996). Purification and characterization of pyrophosphate dependent phosphofructokinase from phosphate-starved *Brassica nigra* suspension cells. Plant Physiol..

[88] Plaxton W.C., Tran H.T. (2011). Metabolic adaptations of phosphate-starved plants. Plant Physiol..

[89] Vengavasi K., Kumar A., Pandey R. (2016). Transcript abundance, enzyme activity and metabolite concentration regulates differential carboxylate efflux in soybean under low phosphorus stress. Indian J. Plant Physi..

[90] Chiou T.J., Aung K., Lin S.-I., Wu C.-C., Chiang S.-F., Su C.-L. (2006). Regulation of phosphate homeostasis by microRNA in Arabidopsis. Plant Cell.

[91] Doerner P. (2008). Phosphate starvation signaling: A threesome controls systemic Pi homeostasis. Curr. Opin. Plant Biol..

[92] Lin W.Y., Lin S.I., Chiou T.J. (2009). Molecular regulators of phosphate homeostasis in plants. J. Exp. Bot..

[93] Shin H., Shin H.-S., Chen R., Harrison M.J. (2006). Loss of *At4* function impacts phosphate distribution between the roots and the shoots during phosphate starvation. Plant J..

[94] Kuo H.F., Chiou T.J. (2011). The role of microRNAs in phosphorus deficiency signaling. Plant Physiol..

[95] Wang T., Zhao M., Zhang X., Liu M., Yang C., Chen Y., Chen R., Wen J., Mysore K.S., Zhang W.H. (2017). Novel phosphate deficiency-responsive long non-coding RNAs in the legume model plant *Medicago truncatula*. J. Exp. Bot..

[96] Liu C., Muchhal U.S., Raghothama K.G. (1997). Differential expression of TPSI1, a phosphate starvation-induced gene in tomato. Plant Mol. Biol..

[97] Burleigh S.M., Harrison M.J. (1998). Characterization of the *Mt4* gene from *Medicago truncatula*. Gene.

[98] Burleigh S.H., Harrison M.J. (1999). The down-regulation of *Mt4*-like genes by phosphate fertilization occurs systemically and involves phosphate translocation to the shoots. Plant Physiol..

[99] Di C., Yuan J., Wu Y., Li J., Lin H., Hu L., Zhang T., Qi Y., Gerstein M.B., Guo Y. (2014). Characterization of stress-responsive lncRNAs in *Arabidopsis thaliana* by integrating expression, epigenetic and structural features. Plant J..

[100] Yuan J., Zhang Y., Dong J., Sun Y., Lim B.L., Liu D., Lu Z.J. (2016). Systematic characterization of novel lncRNAs responding to phosphate starvation in *Arabidopsis thaliana*. BMC Genomics.

[101] Carvalho M.H.C., Sun H., Bowler C., Chua N. (2016). Noncoding and coding transcriptome responses of a marine diatom to phosphate fluctuations. New Phytol..

[102] Griffiths-Jones S., Grocock R.J., van Dongen S., Bateman A., Enright A.J. (2006). miRbase: microRNA sequences, targets and gene nomenclature. Nucleic Acid Res..

[103] Paul S., Datta S.K., Datta K. (2015). miRNA regulation of nutrient homeostasis in plants. Front. Plant Sci..

[104] Nguyen G.N., Rothstein S.J., Spangenberg G., Kant S. (2015). Role of microRNAs involved in plant response to nitrogen and phosphorous limiting conditions. Front. Plant Sci..

[105] Chen X. (2004). A MicroRNA as a translational repressor of APETALA2 in *Arabidopsis* flower development. Science.

[106] Zhang X., Bernatavichute Y.V., Cokus S., Pellegrini M., Jacobsen S.E. (2009). Genome-wide analysis of mono-, di- and trimethylation of histone H3 lysine 4 in *Arabidopsis thaliana*. Genome Biol..

[107] Zhao M., Ding H., Zhu J.K., Zhang F., Li W.X. (2011). Involvement of miR169 in the nitrogen-starvation responses in *Arabidopsis*. New Phytol..

[108] Wang C., Ying S., Huang H., Li K., Wu P., Shou H. (2009). Involvement of *OsSPX1* in phosphate homeostasis in rice. Plant J..

[109] Duan K., Yi K., Dang L., Huang H., Wu W., Wu P. (2008). Characterization of a sub-family of *Arabidopsis* genes with the SPX domain reveals their diverse functions in plant tolerance to phosphorus starvation. Plant J..

[110] Wang C., Huang W., Ying Y., Li S., Secco D., Tyerman S., Whelan J., Shou H. (2012). Functional characterization of the rice SPX-MFS family reveals a key role of OsSPX-MFS1 in controlling phosphate homeostasis in leaves. New Phytol..

[111] Ligaba A., Yamaguchi M., Shen H., Sasaki T., Yamamoto Y., Matsumoto H. (2004). Phosphorus deficiency enhances plasma membrane HC-ATPase activity and citrate exudation in greater purple lupin (*Lupinuspilosus*). Funct. Plant Biol..

[112] Hsieh L.C., Lin S.I., Shih A.C.C., Chen J.W., Lin W.Y., Tseng C.Y., Li W.H., Chiou T.J. (2009). Uncovering small RNA-mediated responses to phosphate deficiency in Arabidopsis by deep sequencing. Plant Physiol..

[113] Liang G., He H., Yu D. (2012). Identification of nitrogen starvation-responsive MicroRNAs in *Arabidopsis thaliana*. PLoS ONE.

[114] Zeng H.Q., Zhu Y.Y., SQ H., Yang Z.M. (2010). Analysis of phosphorus-deficient responsive miRNAs and *cis*-elements from soybean (*Glycine max* L.). J. Plant Physiol..

[115] Zhao X., Liu X., Guo C., Gu J., Xiao K. (2013). Identification and characterization of micro RNAs from wheat (*Triticum aestivum* L.) under phosphorus deprivation. J. Plant Biochem. Biot..

[116] Xu Z., Zhong S., Li X., Li W., Rothstein S.J., Zhang S., Bi Y., Xie C. (2011). Genome wide identification of microRNAs in response to low nitrate availability in maize leaves and roots. PLoS ONE.

[117] Trevisan S., Nonis A., Begheldo M., Manoli A., Palme K., Caporale G., Ruperti B., Quaggiotti S. (2012). Expression and tissue-specific localization of nitrate-responsive miRNAs in roots of maize seedlings. Plant Cell Environ..

[118] Wang Y., Zhang C., Hao Q., Sha A., Zhou R., Zhou X., Yuan L. (2013). Elucidation of miRNAs-mediated responses to low nitrogen stress by deep sequencing of two soybean genotypes. PLoS ONE.

[119] Yan Y., Wang H., Hamera H., Chen X., Fang R. (2014). miR444a has multiple functions in the rice nitrate-signaling pathway. Plant J..

[120] Rogers E.D., Jackson J., Moussaieff A., Aharoni A., Benfey P.N. (2012). Cell types specific transcriptional profiling: Implications for metabolite profiling. Plant J..

[121] Zhang Y.C., Liao J., Li Z., Yu Y., Zhang J., Li Q., Qu L., Shu W., Chen Y. (2014). Genome-wide screening and functional analysis identify a large number of long noncoding RNAs involved in the sexual reproduction of rice. Genome Biol..

